# Thumb Interphalangeal Joint Replacement as an Alternative to Joint Fusion

**DOI:** 10.1155/2020/2961523

**Published:** 2020-04-21

**Authors:** Laurence Grüber, Georges Kohut, Théa Voser

**Affiliations:** Clinique Générale Saint-Anne, Cabinet Kohut. Rue Hans-Geiler 6, Fribourg 1700, Switzerland

## Abstract

Arthrodesis is the generally accepted treatment for symptomatic osteoarthritis of the interphalangeal (IP) joint of the thumb. For 7 thumbs in 5 patients, an IP joint replacement was offered as a pain alleviating and motion conserving treatment option for osteoarthritis. In these cases, an offlabel use of the SR-PIP prosthesis was performed. All patients did well postoperatively with reasonable pain-free range of motion of the interphalangeal joint except one patient who required further surgeries for recurring joint instability. Radiologically, though, an osteolysis around the stems that could lead to loosening of the prosthesis and progressing periarticular ossifications that might cause a blockage of the joint were observed. IP joint arthroplasty represents a viable option on the short term; however, further studies should be conducted to ensure their long-term sustainability.

## 1. Introduction

Osteoarthritis of the interphalangeal (IP) joint of the thumb is not a rare occurrence [1]. When this condition remains symptomatic after conservative management, arthrodesis is the generally admitted treatment [2]. It provides the patient with a stable, nonmoving, but also, pain-free joint. Lin et al. [3] demonstrated in their three-dimensional analysis of the thumb joints in regular daily activities that the spherical grip for a ball with a diameter of 6.3 cm exhibited the largest IP joint flexion with an average of 49°. Twisting a key only needed 22° of flexion. In the literature, for the IP joint, a functional arc of motion between 2° and 43° is considered necessary, when its normal active range of motion is 73° in flexion and 5° in extension [4]. As a result, it would seem logical to try to keep this joint active to maintain the function of the hand as normal as possible and avoid an overload of the metacarpophalangeal (MCP) and carpometacarpal (CMC) joints. Until this day, however, to the best of our knowledge, there have been no studies comparing the dexterity of the hand with the IP joint blocked or kept freely moving. There also is no proof that blocking the IP joint might speed up the development of arthritis in the MCP and CMC joints. Very few reports about IP joint arthroplasty have been described so far [5, 6]. We offered this alternative technique to patients desiring to keep some motion of the IP joint, assuming that they would benefit from it, by reducing pain and improving dexterity in their activities and potentially avoiding an early onset of arthritis in the more proximal joints of the thumb.

### 1.1. Cases Presentation

We report a consecutive case series of 7 arthroplasties of the IP joint of the thumb implanted over a four year period on 5 patients (2 bilateral). Patients presented with a painful degenerative osteoarthritis of the IP joint that was resistant to a well-conducted orthopedic treatment, with concomitant arthritis in the CMC and MCP joints. Patients with jobs that required heavy workloads were not given this option. In addition, we excluded patients whose bone stock was insufficient, as well as thumbs with nonfunctioning tendons. All these patients would clearly benefit more from an arthrodesis. We included 5 subjects who were willing to keep some motion at the tip of the thumb. These patients had to understand that they were offered an offlabel procedure and consent to it. We describe our thumb IP arthroplasty technique with the use of the SR-PIP prosthesis (Stryker). This device consists of titanium, cobalt chrome, and ultrahigh molecular weight polyethylene and is indicated for the replacement of the proximal interphalangeal joint of the long fingers.

For this study, we reviewed all the patients we had previously operated for a thumb IP arthroplasty. They all accepted to come in for medical follow-up consultation. During this meeting, patients underwent a series of questions, a clinical examination, followed by photographic pictures of the hands, and a standard AP and lateral radiographs of the thumb.

During surgery, the joint was accessed by a posterior approach. A Y-shaped incomplete tenotomy of the extensor pollicis longus tendon was performed. Part of the extensor tendon was always spared on the edges of the joint. The collateral ligaments were preserved. Osteophytes were resected on the proximal phalanx. Part of the head of the proximal phalanx was resected by an osteotomy. The medullary canal was enlarged with reamers to adjust the contours for a proper fit of the trial components. Part of the base of the distal phalanx was resected with a burr, in order to accommodate for the volume of the trial component. The medullary canal was prepared in the same manner as for the proximal phalanx. Trial reduction was performed. The size of the prosthesis was chosen so as to allow easy flexion passively, complete extension, and minimal lateral play. Definitive components were cemented. The tendon was repaired using a running absorbable suture. Postoperatively, the thumb was immobilized with a splint for three weeks, maintaining the IP in complete extension. Progressive rehabilitation was then followed.

All our subjects were females. The average age at surgery was 65.9 years. The medium follow-up period was 2.6 years, with a minimum of 18 months. In six of the operated hands, concomitant surgeries were performed. Two underwent a distal interphalangeal (DIP) joint arthrodesis of a long finger. One had three DIP arthrodesis of long fingers. One had an osteophyte resection on the DIP of the index finger. Two had a proximal interphalangeal (PIP) joint arthroplasty of a long finger.

**Figure 1:**
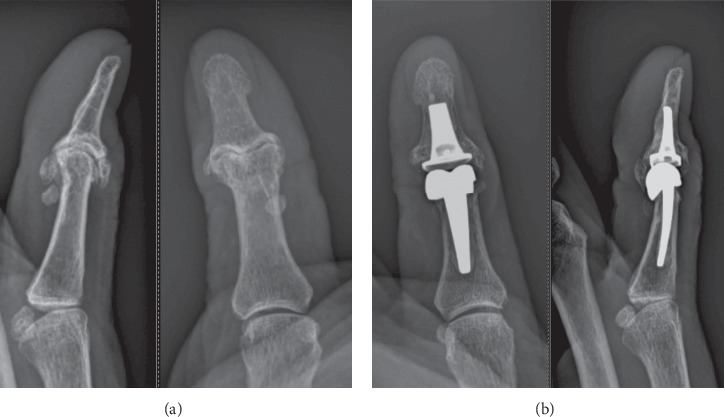
Pre- and postoperative X-rays.

For all patients, X-ray controls were obtained before and after surgery (Figure 1), as well as at the last follow-up. Regrettably, strict AP X-rays of the thumb IP joint were not always obtained before surgery.

### 1.2. Pre- and Postoperative Images

At the last follow-up visit, one patient reported pain exclusively during physical activities. No patient presented pain at rest. No pain was reproduced by direct palpation or mobilization of the joint. The average visual analogue scale (VAS from 0 to 10) of our cohort after the surgery approximates was 0.3, even with activities. It cannot be compared to the preoperative score, as pain level using the VAS has not been asked before surgery.

Medium active range of motion in the IP joint before surgery was 40.8 degrees (information only in 6 cases), while it was 58.1 degrees at the latest follow-up (Table 1). One of the patients showed nearly no mobility. One other presented an impressive range of motion (ROM) of 110°. The global overall mobility of the thumb was good, with an average Kapandji score of 8.9. Postoperatively, the thumb-index (key) pinch strength was at an average of 5.7 kg on the operated thumbs. The same strength was measured on the nonoperated thumbs. It must be emphasized that only 3 measures could be done on thumbs that had not been through a surgery, as 2 patients underwent the surgery on both sides. In comparison, the key pinch of healthy female has been found to be about 7.8 kg on the dominant hand and 8.2 kg on the nondominant hand [7].

One patient needed reoperation. She presented with recurrent dislocation of the joint. It was believed to result from the undersizing of the prosthesis, as it did not recur after replacing it by a larger size at revision. It is important to note that it did not diminish the thumbs overall functioning afterwards. Otherwise, no severe perioperative complications were observed, such as intraoperative fractures, deep infection, or wound contracture.

At the last follow-up, one patient presented with a very limited range of motion in the IP joint. This occurrence was associated with the finding of massive ossifications around the prosthesis. The recognition of growing osteophytes, and ossification, in the substance of the capsule and collateral ligaments was frequent on follow-up X-rays, but had some clinical relevance only in this one case, with one patient presenting with a reduced range of motion. One other patient presented with a significantly high mobility. This situation was not associated with symptoms of instability. No abnormality was seen on the X-rays.

One other observation on successive imaging is the appearance of a radiolucent line around the metaphyseal region of the proximal phalanx prosthesis in two cases. This line could correspond to a stress shielding of the bone in this cemented prosthesis where the cement coating was confined to the stem region on the radiographs. It was first observed at the last follow-up at about two years after surgery.

Two further cases showed a millimetric radiolucent line, surrounded by a sclerotic rim, around the contours of the prosthesis and cement on both the proximal and the distal phalanx. Again, these were first seen at the last follow-up at 3 and 5 years postop. Since no follow-up X-rays were done between the end of the first year of follow-up and the last examination, this line could have appeared at any moment in this time frame. These patients had no symptoms at rest, but one reported pain during regular daily activities. Even though no definition of wear exists regarding finger prosthesis, this appearance makes us suspect this diagnosis, especially in the presence of pain in one patient. As long as the patient experiences only mild discomfort, no further care is required.

Axial deviation could also be an issue. Lack of preoperative AP X-rays of the thumb IP in some cases limits the information available regarding this topic. One case showed an oblique implantation of the stem of the prosthesis on P1, with a valgus of 20° that stayed stable on the follow-up X-rays. Another case showed a preoperative valgus of 18° that was partly corrected with surgery, with 10° persisting postoperatively. One last case presented a 15° valgus deviation that persisted on the postoperative X-rays.

None of our patients complained of instabilities of the replaced IP joint. We did notice, however, in 5 of 7 cases, a tendency for radial angulation of the joint while applying moderate force. The observed angulation was between 2° and 15°. In 4 cases, the joint had a valgus deviation at rest, while in 1 case, the joint had a varus deviation. In 2 cases, a radiolucent line was seen on the X-ray. None of the patients showed ulnar angulation during testing.

Lastly, three of five patients complained of cosmetic discomfort of the thumb. Admittedly, it was often thicker, and in some cases, it presented with a slight valgus deviation. But at present time, all patients would choose to go through the procedure again.

## 2. Discussion

This new technique, described in this article is interesting and seductive. Till this day, only two papers have reported on this subject, both of which were published in April 2019. Schindele et al. [5] reported their results of thumb IP arthroplasty in a 53-year-old patient, presenting with bilateral thumb IP osteoarthritis. She benefited from a silicone arthroplasty on the first side and a surface gliding implant arthroplasty on the second side. Follow-up was for 6 and 4 years, respectively. The patient was satisfied with the outcome. She presented no pain. Active motion on the first side was 35°/0/0° and 25°/0/40° on the second side.

She presented a 20° radial deviation of the IP joint of the thumb with the silicone arthroplasty, and the patient noticed some instability. She was, however, happy with the overall outcome.

McKee et al. [6] reported a 15-year-old trauma patient, who presented with an intra-articular fracture and adjacent laceration at the distal portion of the proximal phalanx of the thumb resulting in near complete loss of the interphalangeal joint after a table saw accident. This patient had a surface gliding implant arthroplasty and a tendon repair. Follow-up was for 22 months. The range of motion of the IP joint was 40°, which can be considered good in this badly injured joint. The patient reported no pain. The DASH score was 6.82.

In our experience though, the implantation of the prosthesis in this location is not a simple procedure, due to the tightness of the soft tissue in this approach. However, in their case report, Schindele et al. [5] did free the radial collateral ligament. This management could simplify the procedure. Consequences on joint stability should be further assessed.

Overall, patients presented a very high satisfaction rate after arthroplasty of the IP joint. They did not report any limitations in everyday activities. It must be emphasized, however, that the reported results of the arthrodesis are evenly good. In the study by Ameline et al., including thumb interphalangeal and finger distal interphalangeal joints arthrodesis, patients presented a VAS of 0.46 [8]. The current literature does not provide any control study comparing the surgical procedure on the IP joint of the thumb, to the orthopedic treatment, not even with the arthrodesis. [9].

Nevertheless, we think that low-demanding activities of daily living, such as small precision tasks, would appear more difficult to patients with arthrodesis and require compensation by the MP and CMC joint. As a result, this might lead to fast evolving arthritis of the CMC joint, especially in people with an MP joint with a limited range of motion. This remains an assumption, however, since as stated earlier, no comparative study has studied the surgical treatment of IP joint arthroplasty yet.

We believe IP arthroplasty has a role in the treatment of arthritis of this joint as it could help patients maintain some motion, and at the same time, it could help with some precision tasks. We understand that the retrospective design of the study, the limited number of cases, as well as a medium follow-up of 2.6 years represent important drawbacks to our study. It remains, though, by far, the largest series reported to date.

The suspected instability observed remains of unknown significance. These symptoms did not in absolute correlate clinically and radiologically. A bigger case load would probably answer our questions. The significance of the radiolucent lines observed on our X-rays also has to be further assessed, as they could mean an evolution towards implant loosening. We had no such severe evolution in our cohort yet. If failure of an implant happened in our patients, an implant removal and secondary arthrodesis should be performed. In this condition, arthrodesis would most probably require a bone graft to compensate with the bone stock loss. Further studies must be carried out, comparing conservative treatment, arthrodesis, and arthroplasty of the IP joint to better assess the outcome and determine the best treatment option in each situation.

## Figures and Tables

**Table 1:** Pre- and postoperative assessments.

Patient	Age (yo) at surgery	Time from surgery (yo)	VAS	ROM IP (°) Preop	ROM IP (°) postop	ROM MCP (°) postop	Kapandji	Pinch arthropl (kg)	Pinch opp (kg)	Reoperation
1	70.4	5	0	30	56	34	9	7.5	5.5	0
2	69.3	2.9	2	55	80	46	9	6	6	0
3	62	2	0	70	110	44	9	3.5	—	0
4	61.7	2.3	0	?	60	38	9	5	—	0
5	73.1	2.5	0	50	10	45	8	6	5.5	0
6	61.8	2.4	0	25	40	64	9	6	—	0
7	62.8	1.5	0	15	51	66	9	6	—	3
Tot	65.9	2.6	0.3	40.8	58.1	48, 1	8.9	5.7	5.7	0.4

## Data Availability

The data used to support the findings of this study are available in Table 1. Radiographs are available from the corresponding author upon request.

## References

[1] Hunter D. J., Zhang Y., Nevitt M. C. (2004). Chopstick arthropathy: the Beijing osteoarthritis study. *Arthritis & Rheumatism*.

[2] Berger A. J., Meals R. A. (2015). Management of osteoarthrosis of the thumb joints. *The Journal of Hand Surgery*.

[3] Lin H.-T., Kuo L.-C., Liu H.-Y., Wu W.-L., Su F.-C. (2011). The three-dimensional analysis of three thumb joints coordination in activities of daily living. *Clinical Biomechanics*.

[4] Hume M. C., Gellman H., McKellop H., Brumfield R. H. (1990). Functional range of motion of the joints of the hand. *The Journal of Hand Surgery*.

[5] Schindele S., Marks M., Herren D. B. (2019). Thumb interphalangeal joint replacements with silicone and surface gliding implants. A case report. *Journal of Hand Surgery (European Volume)*.

[6] McKee D., Domingo-Johnson E. L. (2019). Novel use of joint replacement in a thumb interphalangeal joint. *Case Reports in Orthopedics*.

[7] Puh U. (2010). Age-related and sex-related differences in hand and pinch grip strength in adults. *International Journal of Rehabilitation Research*.

[8] Ameline T., Bégot V., Ardouin L., Hulet C., Hanouz N. (2015). Arthrodesis of thumb interphalangeal and finger distal interphalangeal joints using the intramedullary X-Fuse implant: retrospective analysis of 38 cases. *Chirurgie de la main*.

[9] Spies C. K., Langer M., Hahn P., Müller L. P., Unglaub F. (2018). The treatment of primary arthritis of the finger and thumb joint. *Deutsches Ärzteblatt International*.

